# Challenges in Predicting Intubation in Patients With Severe Coronavirus Disease 2019 (COVID-19) on High-Flow Nasal Cannula: A Focus on Lymphopenia and Conventional Markers

**DOI:** 10.7759/cureus.101700

**Published:** 2026-01-16

**Authors:** Tomotaka Nishizawa, Tsuyoshi Shiga, Masako Amano, Hidekazu Matsushima

**Affiliations:** 1 Pulmonology, Research Institute of the McGill University Health Centre, Montreal, CAN; 2 Clinical Pharmacology, Jikei University School of Medicine, Tokyo, JPN; 3 Respiratory Medicine, Saitama Red Cross Hospital, Saitama, JPN

**Keywords:** covid-19, high-flow nasal cannula, intubation, lymphocytes, risk factors

## Abstract

Background

Intubation risk assessment in severe coronavirus disease 2019 (COVID-19) remains a key clinical challenge, especially for patients treated with high-flow nasal cannula (HFNC). We aimed to clarify whether lymphopenia at admission is associated with the subsequent need for intubation.

Methods

This retrospective study included all consecutive COVID-19 patients admitted to Saitama Red Cross Hospital in Saitama, Japan, from July to September 2021, who required HFNC therapy at presentation. Intubation was performed if the saturation of peripheral oxygen (SpO₂) remained below 90% despite HFNC with a fraction of inspired oxygen (FiO₂) of 80%. We compared clinical and laboratory characteristics between patients who required intubation and those successfully managed on HFNC to identify prognostic factors present at the time of HFNC initiation.

Results

Out of 49 analyzed patients, 15 required intubation during hospitalization and 34 did not. Age and body mass index (BMI) were similar between groups. The Mann-Whitney U tests showed significantly lower lymphocyte counts (intubation: 739.1/μL (interquartile range (IQR) 516.3) vs. non-intubation: 909.0/μL (IQR 423.4); p=0.034), lower lymphocyte percentage (8.0% (IQR 13.0) vs. 15.6% (IQR 9.6); p=0.037), and longer hospital stays (17 days (IQR 18) vs. 14 days (IQR 10); p=0.007) in the intubation group. Multivariate stepwise logistic regression identified lower lymphocyte count (OR 0.997; 95% CI: 0.995-1.000; p=0.048) and longer hospital stay (OR 1.09; 95% CI: 1.01-1.20; p=0.036) as independent indicators of intubation.

Conclusion

In severe COVID-19 patients treated with HFNC, admission lymphopenia was independently associated with subsequent intubation; however, its discriminative performance as a prognostic marker was modest. Lymphocyte counts may aid prognostic risk stratification when interpreted cautiously alongside conventional clinical markers and oxygenation indices, particularly in the context of current and future respiratory viral outbreaks.

## Introduction

The novel coronavirus disease, coronavirus disease 2019 (COVID-19), emerged suddenly in mid-December 2019 and has since rapidly spread worldwide [1]. COVID-19 is often severe enough for older patients, obese patients, or those with health problems such as hypertension or diabetes to require oxygen supplementation [2-6]. Moreover, older age, high body mass index (BMI), and other health conditions are risk factors for severe COVID-19 [2-4]. The efficacy of high-flow nasal cannula (HFNC) oxygen therapy [7] in patients with COVID-19 is still unclear, given that HFNC therapy promotes recovery in some patients, whereas others do not respond sufficiently and require intubation for oxygenation and disease management.

Identifying and evaluating the relevant factors and devising appropriate management strategies would reduce the complications of COVID-19 disease by facilitating early diagnosis and treatment [2]. However, the factors most useful for detecting, at the time of diagnosis, which patients are likely to deteriorate and ultimately require intubation, particularly among those managed initially with HFNC, are not fully understood. In particular, lymphopenia at the initial consultation may provide prognostic insight and help clinicians judge whether a patient with COVID-19 should be admitted directly to the intensive care unit (ICU) in the anticipation of probable intubation or can be managed in a general ward with HFNC therapy.

In this study, the aim was to evaluate, in a retrospective cohort of COVID-19 patients treated with HFNC, how clinical variables at admission, including lymphopenia, are associated with the subsequent need to escalate from HFNC to invasive mechanical ventilation. To this end, patients who successfully avoided intubation and were discharged after being weaned from HFNC were compared with those in whom HFNC was insufficient and who subsequently required intubation, in order to clarify prognostic factors present at the time of HFNC initiation and to provide hypothesis-generating insights that may inform bedside decision-making.

Now that the acute phase of the pandemic has passed, it is essential to clarify the specific added value of this study. Focusing on severe COVID‑19 patients requiring HFNC and on the association between admission lymphopenia and subsequent intubation may still be clinically relevant for risk stratification and resource allocation, not only in the current era but also in the context of future respiratory viral outbreaks.

## Materials and methods

Study design and patients

This retrospective study involved patients with early respiratory failure at Saitama Red Cross Hospital in Saitama, Japan, and evaluated the available data in the patients' medical records. We studied prognostic risk factors associated with the need for progression to intubation for oxygen supplementation after HFNC therapy for severe COVID-19 disease. The study was approved by the Ethics Committee of Saitama Red Cross Hospital (approval number: 21-T; date: September 10, 2021). The requirement for written informed consent was waived because we evaluated de-identified retrospective data only [2]. Instead of written informed consent, this research used an opt-out consent model, which meant that, at any time, patients could choose to discontinue their participation and have their information deleted from the registry [2]. We collected the medical records of laboratory-confirmed hospitalized cases of COVID-19 between July 1, 2021, and September 30, 2021. During this period, most cases were caused by the Delta variant. Vaccination had been initiated for healthcare workers, but the vaccine supply was still insufficient for the general population. Patients who received HFNC therapy beginning at the time of consultation also were included in this study. COVID-19 diagnoses were confirmed by real-time reverse-transcription polymerase chain reaction (PCR) assay of nasal or pharyngeal swab specimens [2]. Salivary PCR analysis was not used to diagnose any patients. During the study period, all consecutive patients meeting these criteria and requiring HFNC as initial advanced respiratory support were included to minimize selection bias. This was a single-center retrospective study that included all consecutive patients who met the eligibility criteria and required HFNC during a predefined three‑month period. Because the sample size was determined by case availability rather than by an a priori calculation, no formal sample size estimation was performed, and the analyses should be interpreted in light of this constraint.

Clinical management and intubation criteria

In Japan, COVID‑19 severity is classified into four categories (mild, moderate I, moderate II, and severe). Moderate II is defined as respiratory insufficiency requiring supplemental oxygen (saturation of peripheral oxygen (SpO₂) <93% on room air), whereas severe disease corresponds to respiratory failure requiring mechanical ventilation or ICU admission. We restricted our analysis to patients classified within the severe category. All patients were managed with HFNC as initial advanced support. Intubation was mandated if SpO₂ could not be maintained ≥90% even when high-flow parameters delivered a fraction of inspired oxygen (FiO₂) of 80%. In clinical practice, when SpO₂ values were trending toward 90% under FiO₂ of 80% and global assessment indicated likely deterioration, clinicians were advised to consider early intubation rather than waiting for more profound desaturation. Decisions were based on continuous clinical monitoring following the established respiratory guidelines. In our hospital, treatment strategies, including the use of HFNC, admission to the ICU, and pharmacologic therapies, were determined in accordance with these guideline‑based severity categories and the availability of treatments at that time. For the present analysis, we focused on severe patients who required HFNC, and we did not include patients managed solely with conventional oxygen therapy.

Data collection

Medical data regarding patient demographics, summarized medical histories, vital signs, outpatient medications, chest radiographs, and laboratory results at diagnosis were collected. In addition, data regarding respiratory support (HFNC therapy, positive-pressure ventilation after intubation) and the total number of days of oxygen administration were collected [2]. We defined severe respiratory illness in the setting of COVID-19 as that requiring either HFNC therapy or intubation for oxygen supplementation [2].

Statistical analysis

Continuous variables were compared by the Mann-Whitney U test. Logistic regression with stepwise (the Akaike Information Criterion (AIC)-based) selection included variables of clinical relevance and univariate significance (p<0.10). Statistical significance was assigned at p<0.05. We referred to previous studies [8-11] to confirm that the selection of these variables was appropriate. All statistical analyses were conducted using GraphPad Prism (version 10.0; GraphPad Software Inc., La Jolla, CA, USA). Model fit was assessed using the AIC. In the multivariable logistic regression model including lymphocyte count and hospital stay, the final model had an AIC of 55.71. Given that the ratio of sample size to parameters (n/k) was 16.3, we also calculated the corrected AIC (AICc=56.25), which did not alter the model selection. To reduce the risk of overfitting in this relatively small cohort, we restricted the number of covariates in the multivariable model, prioritizing variables with established clinical relevance and those showing at least a weak association in univariable analyses (p<0.10). The primary objective of the modelling was to explore prognostic associations rather than to construct a definitive predictive tool.

## Results

Baseline characteristics of the HFNC and intubated groups

A total of 49 patients with severe or very severe COVID-19 who received HFNC therapy during the study period were included in the analysis. The HFNC group comprised 34 patients, and the intubation group included 15 patients (Table 1). No significant differences were observed between groups regarding age (HFNC group: 52 years (interquartile range (IQR) 14); intubation group: 51 years (IQR 15); p=0.88) or BMI (HFNC group: 24.8 kg/m² (IQR 7.0); intubation group: 24.9 kg/m² (IQR 10.9); p=0.97) (Table 1). Men accounted for 29 cases in the HFNC group and 11 cases in the intubation group. Among all survivors, consciousness was preserved; only one non-survivor (from the intubation group) presented with altered mental status in response to verbal stimuli. Vaccination rates were low, with three patients in the HFNC group having received one vaccine dose prior to admission and none in the intubation group. The groups did not differ significantly in major demographic characteristics.

**Table 1:** Patient characteristics, blood tests, and length of hospital stay with univariate comparison between the intubation and HFNC groups (Mann-Whitney U test) Median (IQR) values; only lymphocyte metrics and length of stay were significantly different. BMI: body mass index; WBC: white blood cell; CRP: C-reactive protein; LDH: lactate dehydrogenase; IQR: interquartile range; HFNC: high-flow nasal cannula

Variable	Intubation group (n=15)	HFNC group (n=34)	U-statistic	P-value
Lymphocyte count (/μL)	739.1 (483.7-1000.0)	909.0 (616.1-1039.5)	139.00	0.034
Lymphocyte fraction (%)	8.0 (3.0-16.0)	15.6 (11.1-20.7)	126.00	0.037
Age (years)	51 (47-62)	52 (45-59)	200.00	0.88
BMI (kg/m²)	24.9 (21.8-32.7)	24.8 (22.0-29.0)	187.00	0.97
WBC (/μL)	6250 (4837-11840)	5915 (4740-8440)	200.00	0.47
CRP (mg/dL)	8.3 (3.97-16.2)	6.9 (3.9-9.3)	201.00	0.48
Ferritin (ng/mL)	699 (381-2717)	917 (353-1740)	156.00	0.45
LDH (U/L)	488 (405-785)	455 (376-602)	184.50	0.52
D-dimer (μg/mL)	0.7 (0.5-3.7)	0.5 (0.5-0.7)	225.00	0.63
Length of hospital stay (days)	17 (14-32)	14 (11-21)	264.50	0.007

Hematological findings at hospital admission

At admission, white blood cell (WBC) count and concentrations of serum C-reactive protein (CRP), ferritin, lactate dehydrogenase (LDH), and D-dimer did not differ significantly between the two groups. Specifically, median WBC counts were 5915/μL (IQR 3700) (HFNC) versus 6250/μL (IQR 7003) (intubation) (p=0.47). CRP was 6.9 mg/dL (IQR 5.4) (HFNC) and 8.3 mg/dL (IQR 12.23) (intubation) (p=0.48). Ferritin and LDH also showed no significant differences (p=0.45 and p=0.52, respectively). However, lymphocyte parameters were significantly lower in the intubation group: the lymphocyte count was 739.1/μL (IQR 516.3) in the intubation group compared to 909.0/μL (IQR 423.4) in the HFNC group (p=0.034). Lymphocyte fraction mirrored this trend: 8.0% (IQR 13.0) in the intubation group versus 15.6% (IQR 9.6) in the HFNC group (p=0.037). D-dimer levels were not significantly different between the two groups (0.7 μg/mL (IQR 3.2) vs. 0.5 μg/mL (IQR 0.2) (p=0.63) (Table 1).

Length of hospital stay

The length of hospital stay was significantly longer in the intubation group (17 days (IQR 18)) than in the HFNC group (14 days (IQR 10) (p=0.007). Only one patient from the intubation group died due to worsening respiratory failure during the study period (Table 1).

Treatments

All patients, with or without later intubation, received standard care with high-dose corticosteroid therapy and heparin anticoagulation (Table 2). High-dose corticosteroid therapy in this study consisted primarily of intravenous methylprednisolone 500 mg daily for the first three days. Thereafter, the dose was tapered as early as clinically feasible based on the patient's condition, with the aim of complete discontinuation as soon as safely possible. Furthermore, the other treatment approaches were also nearly identical between the two groups. All 15 patients (100%) in the intubation group received both remdesivir and baricitinib, and almost all patients in the HFNC group were treated with these agents, with 32 of 34 (94.1%) receiving remdesivir and 33 of 34 (97.1%) receiving baricitinib (Table 2). Treatment with corticosteroids and remdesivir was initiated immediately after admission, and baricitinib was also administered early in the clinical course, which indicates that these treatments were given before intubation in the intubation group.

**Table 2:** Treatments administered during hospitalization in the intubation and HFNC groups HFNC: high-flow nasal cannula

Treatment	Intubation group (n=15)	HFNC group (n=34)
High-dose steroid therapy	15 (100%)	34 (100%)
Heparin	15 (100%)	34 (100%)
Remdesivir	15 (100%)	32 (94.1%)
Baricitinib	15 (100%)	33 (97.1%)

Logistic regression analysis for factors predictive of intubation

Univariate analyses identified lower lymphocyte count (p=0.034), lower lymphocyte fraction (p=0.037), and a longer hospital stay (p=0.007) as significant predictors for intubation during HFNC therapy. Lymphocyte count yielded an odds ratio (OR) of 0.997 (95% CI: 0.995-1.00) per unit increase; hospital stay yielded an OR of 1.14 (1.04-1.26) per day. In multivariate logistic regression analysis, lymphocyte count on admission (OR 0.997; 95% CI: 0.995-1.000; p=0.048) and length of hospital stay (OR 1.09; 95% CI: 1.01-1.20; p=0.036) persisted as independent predictors of intubation (Table 3). Neither D-dimer nor other hematologic/inflammatory markers retained statistical significance in the multivariable model (Table 3). The final multivariable model including lymphocyte count and hospital stay had an AIC of 55.71 (AICc=56.25), and the use of AICc did not change the set of selected predictors.

**Table 3:** Logistic regression analysis regarding the need for intubation Bold indicates variables retained as independent predictors in the multivariate model. Variables retained in the final model after stepwise AIC-based selection are shown in bold. *Univariate p-values for continuous variables are from the Mann-Whitney U tests. BMI: body mass index; WBC: white blood cell; CRP: C-reactive protein; LDH: lactate dehydrogenase; AIC: Akaike Information Criterion

Variable	Univariate p-value*	Univariate OR (95%CI)	Multivariate OR (95%CI)	Multivariate p-value
Age (years)	0.88	1.00 (0.95-1.06)	-	-
BMI (kg/m²)	0.97	0.98 (0.88-1.09)	-	-
WBC (/μL)	0.47	1.00 (1.00-1.00)	-	-
Lymphocyte count (/μL)	0.034	0.997 (0.995-1.00)	0.997 (0.995-1.000)	0.048
Lymphocyte fraction (%)	0.037	0.93 (0.84-1.03)	-	-
CRP (mg/dL)	0.48	1.03 (0.96-1.11)	-	-
Ferritin (ng/mL)	0.45	1.00 (1.00-1.00)	-	-
LDH (U/L)	0.52	1.00 (0.99-1.01)	-	-
D-dimer (μg/mL)	0.63	1.03 (0.91-1.18)	-	-
Hospital stay (days)	0.007	1.14 (1.04-1.26)	1.09 (1.01-1.20)	0.036

## Discussion

Our study, which focuses exclusively on severe COVID‑19 patients initially managed with HFNC, suggests that lower admission lymphocyte counts are prognostically associated with subsequent intubation, even after adjustment for key clinical covariates such as age, BMI, inflammatory markers, and D‑dimer. However, given the small sample size and the observational, single‑center design, this association should be regarded as exploratory and hypothesis‑generating rather than definitive. In our multivariable analysis, lower lymphocyte count was statistically associated with subsequent intubation. However, the upper bound of the 95% confidence interval for the OR approached 1.000, indicating only borderline statistical significance. Given the small sample size and this narrow confidence interval bordering on the null value, the observed association should be interpreted as suggestive but potentially imprecise, and our findings should be regarded as exploratory rather than definitive.

Intubation timing in this study was standardized by using SpO₂ <90% at an FiO₂ of 80% on HFNC as the trigger, thereby ensuring consistency and reducing clinician-dependent variability across cases. Furthermore, the analysis indicates that lymphocyte count, a widely available and low-cost laboratory parameter, was a better single marker for subsequent intubation than D-dimer, suggesting that its potential utility in risk stratification may have been underestimated relative to traditional coagulation markers in the triage of severe COVID-19 [8-11]. Lymphopenia is a common hematologic abnormality in COVID-19 and has been consistently associated with disease severity, need for invasive mechanical ventilation, and mortality across multiple cohorts. Several large observational studies have demonstrated that patients with low lymphocyte counts at presentation are more likely to require ICU admission or intubation and have higher short-term and in-hospital mortality, suggesting that lymphopenia reflects a state of immune dysfunction and impaired antiviral defense. Mechanistically, severe acute respiratory syndrome coronavirus 2 (SARS-CoV-2)-associated lymphopenia may result from direct viral effects on lymphocytes, cytokine-mediated apoptosis, and disruption of lymphopoiesis and therefore identifies patients at increased risk of secondary infection and multi-organ failure who may benefit from earlier critical care consultation and closer monitoring [12-16].

In this study, we limited the diagnosis to severe COVID-19 on admission and examined predictors of intubation. Neither age, nor BMI, nor other likely poor prognostic factors differed between our patient groups. The prolonged hospital stay might reflect complications associated with intubation rather than those from COVID-19 itself. Therefore, we believe that it is unnecessary to immediately intubate all patients with severe COVID-19. In addition, we consider that it is important to assess other prognostic factors and to monitor the progress of HFNC and various drug therapies in patients who have severe COVID-19 but who are at lower risk of intubation. One particular benefit of HFNC is that the patient is not heavily sedated and therefore can be managed in a general ward. These strategies may help to shorten the hospital stay among patients with severe COVID-19.

Lymphocyte counts may aid prognostic risk stratification when interpreted cautiously alongside conventional clinical markers and oxygenation indices, particularly in the context of ongoing viral evolution and changing treatment practices. In this regard, incorporating lymphocyte count into a multimodal assessment framework may help refine decisions regarding ICU admission, the escalation of respiratory support, and the timing of intubation while avoiding overreliance on any single parameter. Such an approach could remain informative not only for COVID‑19 but also for future waves of SARS‑CoV‑2 and other emerging respiratory viral infections that share similar patterns of virus‑induced lymphocyte dysregulation.

Limitations

This study has several limitations. First, the sample size was relatively small, which constrains the statistical power and increases the imprecision of the effect estimates; therefore, the findings should be interpreted as exploratory and hypothesis‑generating rather than definitive. Second, the study was conducted in a single center in Japan over a defined three‑month enrollment period, which may limit the generalizability of the results to other institutions or healthcare systems. Third, the decision to proceed with tracheal intubation, while guided by a tightly standardized institutional protocol based primarily on SpO₂ under high FiO₂, ultimately remained a pragmatic clinical judgment at the bedside, which may have introduced variability in the timing of intubation across patients. In addition, there was substantial overlap in lymphocyte counts between patients who did and did not require intubation, which limits the clinical usefulness of any simple lymphocyte cut‑off for individual decision‑making. Finally, although we adjusted for several clinically relevant covariates, including age, BMI, major comorbidities, inflammatory markers, and D‑dimer, residual confounding from unmeasured or incompletely measured factors cannot be excluded.

## Conclusions

In severe COVID-19 patients on HFNC, admission lymphopenia was independently associated with subsequent intubation for refractory hypoxemia, although its overall predictive value appeared limited. Protocolized intubation thresholds and comprehensive laboratory assessment suggested that immune status may serve as a supportive adjunct rather than a standalone clinical decision tool. Further multicenter studies are needed to clarify the pathobiological roles of lymphocytes and to refine their contribution to evolving COVID-19 risk stratification.

## References

[1] Liang W, Liang H, Ou L (2020). Development and validation of a clinical risk score to predict the occurrence of critical illness in hospitalized patients with COVID-19. JAMA Intern Med.

[2] Goto N, Wada Y, Ikuyama Y (2021). The usefulness of a combination of age, body mass index, and blood urea nitrogen as prognostic factors in predicting oxygen requirements in patients with coronavirus disease 2019. J Infect Chemother.

[3] Gao YD, Ding M, Dong X (2021). Risk factors for severe and critically ill COVID-19 patients: a review. Allergy.

[4] Hernández-Galdamez DR, González-Block MÁ, Romo-Dueñas DK, Lima-Morales R, Hernández-Vicente IA, Lumbreras-Guzmán M, Méndez-Hernández P (2020). Increased risk of hospitalization and death in patients with COVID-19 and pre-existing noncommunicable diseases and modifiable risk factors in Mexico. Arch Med Res.

[5] Rashedi J, Mahdavi Poor B, Asgharzadeh V (2020). Risk factors for COVID-19. Infez Med.

[6] Huang C, Wang Y, Li X (2020). Clinical features of patients infected with 2019 novel coronavirus in Wuhan, China. Lancet.

[7] Venanzi A, Di Filippo P, Santagata C, Di Pillo S, Chiarelli F, Attanasi M (2022). Heated humidified high-flow nasal cannula in children: state of the art. Biomedicines.

[8] AlSamman M, Caggiula A, Ganguli S, Misak M, Pourmand A (2020). Non-respiratory presentations of COVID-19, a clinical review. Am J Emerg Med.

[9] Aghili SM, Ebrahimpur M, Arjmand B (2021). Obesity in COVID-19 era, implications for mechanisms, comorbidities, and prognosis: a review and meta-analysis. Int J Obes (Lond).

[10] Du Y, Lv Y, Zha W, Zhou N, Hong X (2021). Association of body mass index (BMI) with critical COVID-19 and in-hospital mortality: a dose-response meta-analysis. Metabolism.

[11] Zhao X, Gang X, He G, Li Z, Lv Y, Han Q, Wang G (2020). Obesity increases the severity and mortality of influenza and COVID-19: a systematic review and meta-analysis. Front Endocrinol (Lausanne).

[12] Hastak P, Cromer D, Malycha J (2024). Defining the correlates of lymphopenia and independent predictors of poor clinical outcome in adults hospitalized with COVID-19 in Australia. Sci Rep.

[13] Kılıc J, Ebik B, Bacaksız F, Ekin N, Kalın BS (2023). Is lymphopenia a predictor of mortality in patients with COVID-19?. Acta Clin Croat.

[14] Yadava K, Sichelstiel A, Luescher IF, Nicod LP, Harris NL, Marsland BJ (2013). TSLP promotes influenza-specific CD8+ T-cell responses by augmenting local inflammatory dendritic cell function. Mucosal Immunol.

[15] Karimi A, Shobeiri P, Kulasinghe A, Rezaei N (2021). Novel systemic inflammation markers to predict COVID-19 prognosis. Front Immunol.

[16] Michels EH, Appelman B, de Brabander J (2024). Host response changes and their association with mortality in COVID-19 patients with lymphopenia. Am J Respir Crit Care Med.

